# Situs Inversus Totalis in Association With Duodenal Atresia

**DOI:** 10.7759/cureus.17764

**Published:** 2021-09-06

**Authors:** Murtadha A Alshaikh, Hussain A Al Ghadeer, Hamed Alabad, Madinah Almohsin, Roqaia A Al Ali

**Affiliations:** 1 Pediatric Department, Maternity and Children Hospital, Al-Ahsa, SAU; 2 Urology Department, Defense Force Hospital, Manama, BHR; 3 Pediatric Surgery Department, Maternity and Children Hospital, Al-Ahsa, SAU; 4 Neonatology Department, Maternity and Children Hospital, Al-Ahsa, SAU

**Keywords:** situs inversus totalis, duodenal atresia, duodenostomy, malrotation, situs inversus with dextrocardia

## Abstract

Situs inversus totalis is the mirror image transposition of the abdominal-thoracic viscera. Approximately one in every 5,000 to 20,000 live births has situs inversus totalis. Most commonly, it is found incidentally and is asymptomatic. A number of malformations, including cardiac, splenic, and gastrointestinal, have been associated with this condition. Coexistence with duodenal atresia is extremely rare, reported in fewer than 30 cases worldwide and one case in Saudi Arabia. We report a preterm neonate who presented with bilious vomiting. Diagnosis of situs inversus totalis with duodenal atresia type III was established and other anomalies were ruled out. The patient was managed surgically by duodenal-duodenostomy and Ladd’s procedure. The report emphasizes the importance of identifying this condition and recognizing the "mirror anatomy" before carrying out an operation. Once the diagnosis is confirmed, surgical intervention must be performed as soon as possible to prevent complications.

## Introduction

Situs inversus disorder is described as a spectrum of transposition of the internal body organs which can be complete (totalis) where both the thoracic and abdominal organs are reversed, resulting in a mirror image of the normal body anatomical organs or might be partial (partialis) where either the thoracic or abdominal organs are reversed [1].

Other associated congenital defects, such as congenital heart disease, splenic malformation, and intestinal and biliary atresia, can worsen situs inversus totalis [2,3]. Situs inversus totalis is most commonly asymptomatic and is discovered by chance during laparotomy or radiographic investigations. However, when an intestinal obstruction like a midgut volvulus or atresia is associated with situs inversus totalis, the patient will likely present early in the neonatal period [4,5].

In Saudi Arabia, only one neonatal case was reported as situs inversus totalis and duodenal atresia [6]. We are presenting a case of a 34-weeker baby girl with situs inversus totalis and duodenal atresia and emphasize the importance of diagnosing such cases prior to surgical correction.

## Case presentation

This is a case of a baby girl, delivered at a gestational age of 34 weeks via an emergent cesarean section on account of fetal distress. The mother is a Saudi woman aged 37, who was a gravida 4, para 2, with two abortions. The pregnancy was described as uneventful. The infant was delivered through thin meconium-stained amniotic fluid, cried immediately, and was given an Apgar score of 7 and 7 at one and five minutes, respectively. She had a birth weight of 1660 grams, lies at 10th percentile for age and gestation, length of 39.5 cm at 4th percentile, and head circumference of 30 cm at 33rd percentile. The vital signs were within normal ranges for age. She was active, pink in color, and not dysmorphic. An examination of the respiratory system revealed increased respiratory effort, subcostal retraction, and nasal flaring, for which she was placed on non-invasive ventilation and 35% fractionated oxygen (FiO2). Additionally, a cardiac exam revealed heart sounds and palpable impulses over the right precordium. Another systemic examination was within normal limits for age. The patient was shifted to a level three neonatal intensive care unit (NICU) after stabilization.

**Figure 1:**
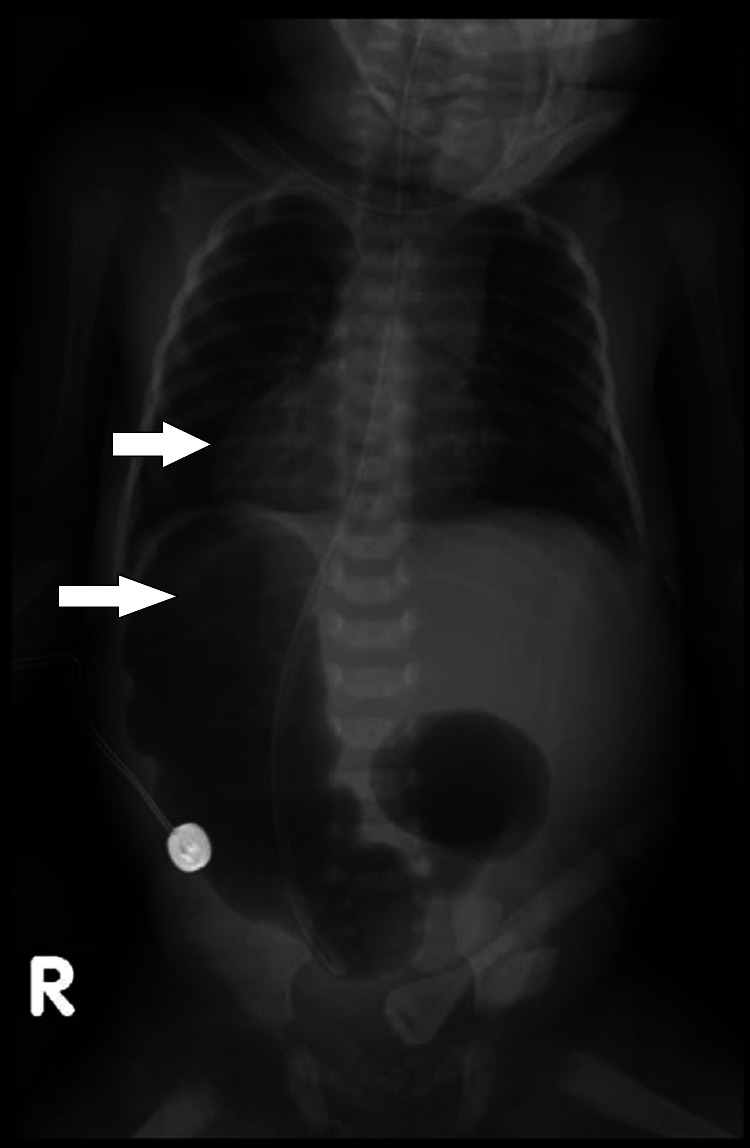
Plain X-ray of chest and abdomen revealing right-sided cardiac shadow, NGT in the stomach with double bubble sign on the right side, and hepatic shadow on the left side. A temperature probe on the right side of the abdomen was used as a site marker. NGT, frequent nasogastric tube.

On her first day of life, she developed persistent green gastric aspirates, for which she had serial radiographs taken (Figure 1). Radiological findings identified dextrocardia, a situs inversus, and probable duodenal atresia. Both ECGs and echocardiograms have confirmed the diagnosis of dextrocardia and normal cardiac anatomy. As well, ultrasonography has showed inversed abdominal organs but otherwise normal anatomy. Similarly, brain ultrasonography displayed no congenital anomalies during the screening process. Further, laboratory investigations including renal and liver function tests all fell within normal limits for her age and she had a normal female karyotype on chromosomal analysis.

**Figure 2:**
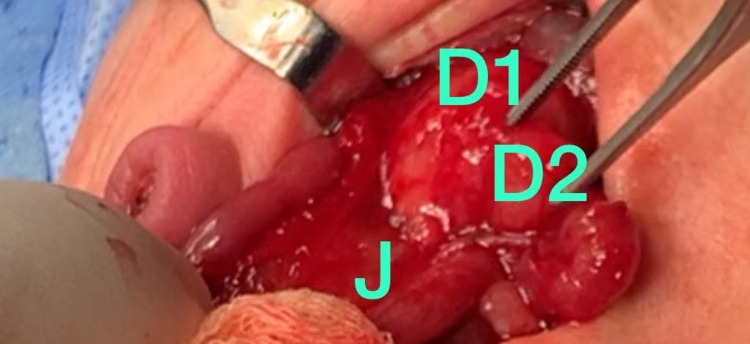
A picture during laparotomy showed type III duodenal atresia complete obstruction at the end of the second part of the duodenum. D1: first part of duodenum; D2: second part of duodenum; J: jejunum.

**Figure 3:**
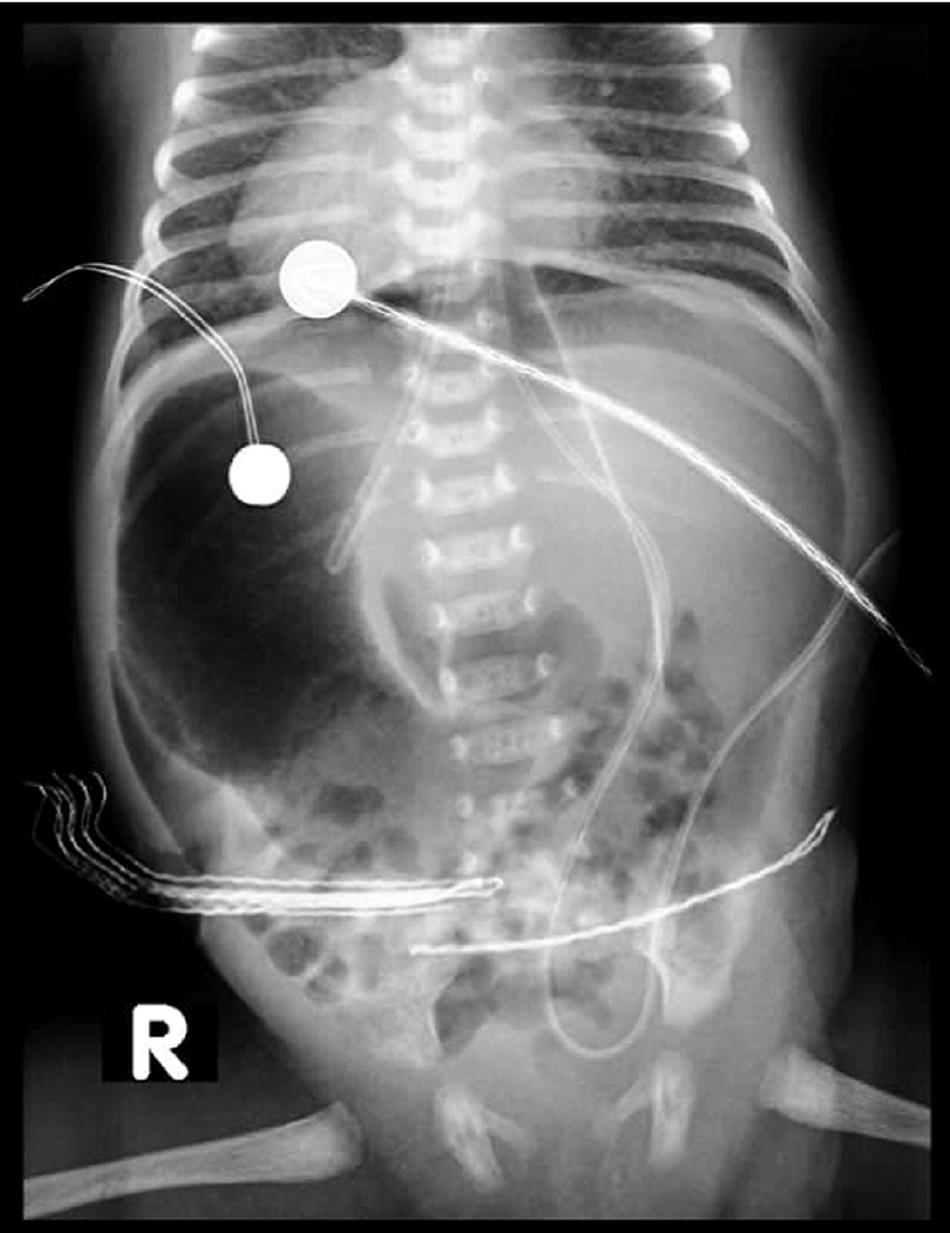
Abdomen postoperative X-ray showed megaduodenum and pneumoperitoneum.

**Figure 4:**
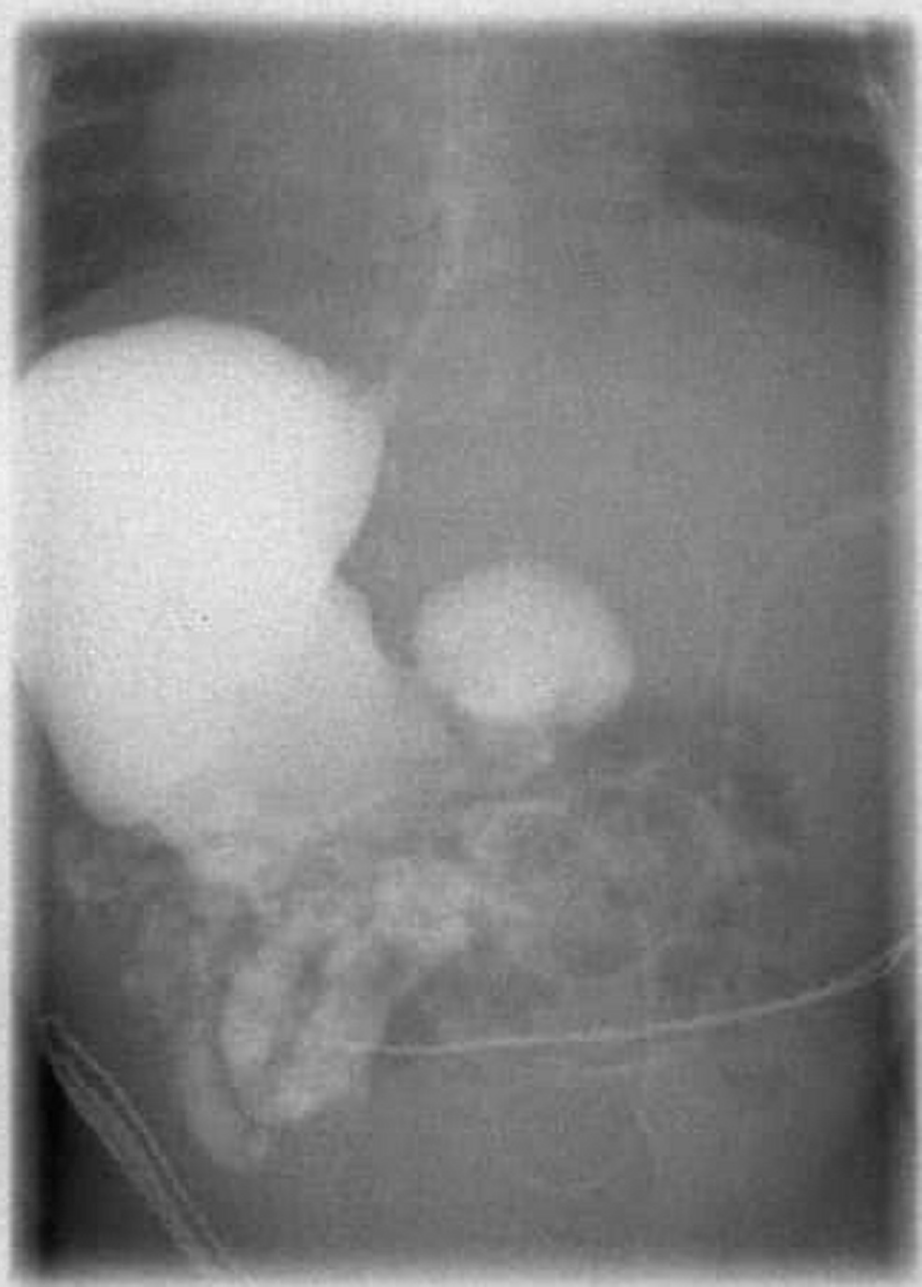
Two weeks postoperative upper GI contrast study showed no leakage from anastomosis area with megaduodenum. GI, gastrointestinal.

Side-to-side duodenal-duodenostomy and Ladd's procedure were performed on her third day of life. An intraoperative examination revealed right-sided stomach and spleen, left-sided liver, and type III complete duodenal atresia, where the two ends of the duodenum separate distal to the ampulla of Vater, along with intestinal malrotation (Figure 2). The patient had a stable course immediately after the operation. Nevertheless, on the second postoperative day, abdominal distension and pneumoperitoneum developed, suggesting an anastomotic leak (Figure 3). An abdominal Penrose drain was inserted and kept in place for two weeks with a frequent nasogastric tube (NGT) suctioning until the abdominal free air was resolved completely. An upper gastrointestinal (GI) contrast study at that time showed normal findings and did not indicate any leakage from the area of anastomoses (Figure 4). Feeding was thus started, and she demonstrated a good tolerance for the food.

The infant made an uneventful recovery and was discharged home to care of parents after 40 days, at corrected age of 40 weeks and 5 days. During outpatient follow-ups, the patient demonstrated good overall health, nutrition, bowel habits, growth catch-up, and normal neurodevelopmental milestones. Currently, she is seven months old, feeding breast milk and high-energy milk formula. In light of the inheritance pattern, she was referred to a higher center for further genetic testing.

## Discussion

A situs inversus is an inverted position of abdominal and thoracic organs. A situs inversus totalis occurs when the visceral organs are completely transposed (mirror image of their normal positioning) as in our case. It is called situs inversus partialis when the inversion occurred solely in the abdominal organs. Normal positioning of the organs as observed in most individuals is called situs solitus [1]. It was Fabricius who first described situs inversus as a mirror image of the normal thoracic and abdominal anatomy [7]. There is approximately one in every 5000 to 20,000 live births with situs inversus totalis [1]. Situs inversus totalis can either be isolated or coexist with other birth defects. The exact etiology of situs inversus is not fully understood [8]. However, it has been proposed that it might be related to the rotations of the cardiac tube during organogenesis and that any mechanical disturbance arising from any of the rotational movements can cause the heart to be positioned incorrectly or result in dextrocardia, though the underlying mechanism is still a mystery [9]. Situs inversus totalis tends to be autosomal recessive, but it can also be autosomal dominant or X-linked [10]. Multiple inheritance patterns are suggested by genetic susceptibility and familial occurrence.

Ciliary abnormalities are the most common association with situs inversus totalis (the primary ciliary dyskinesia disorders). Patients with primary ciliary dyskinesia (PCD)/Kartagener syndrome have situs inversus totalis in about 50% of the cases [11]. The association of situs inversus and duodenal atresia has been described in fewer than 30 patients [12,13]. In theory, a failure to recanalize could lead to duodenal atresia [14]. Gray and Skandalakis classified duodenal atresia into three types: type I, where mucosa and submucosa form a web without a defect in the muscle coat. If this web is thin, windsock deformity can occur. The base of the membrane will be in the second part of the duodenum. In type II duodenal atresia, the atretic duodenal end is connected by a fibrous cord that is separated by some distance with an intact mesentery. Also, type III in which the atretic duodenal end is separated by some distance but without any tissue intervening and the mesentery has a V-shaped defect as was the case in our patient [15].

It is estimated that situs inversus totalis patients have a congenital heart disease incidence of 5% to 10%, the majority of which is a ventricular septal defect, transposition of the great vessels, and valvular disease [16]. Annular pancreas, biliary atresia, preduodenal portal vein, diaphragmatic hernia, renal dysplasia, splenic malformation, ectopic thyroid, meconium ileus, lung fibrosis, ear, eye, and vertebral deformities are some of the other related anomalies [2].

When fetal situs inversus is detected via prenatal ultrasound, additional testing such as fetal magnetic resonance imaging (MRI) or fetal echocardiography can be performed to help understand the fetus' developmental stage. This could affect management and prognosis. Following birth, additional imaging tests are also recommended to determine whether there are any associated abnormalities. In some studies, ultrasonography is suggested as a radiographic modality of choice to avoid incorrect labeling on radiographs and to assess duodenal atresia [17]. In our patient, a postnatal examination was conducted with these considerations in mind. There are no malformations associated with this case, rather than duodenal atresia.

Identifying situs inversus in the body is important for diagnosing medical problems and preventing surgical errors that can occur when reversed anatomy goes unnoticed. Duodenal atresia (DA) and malrotation require surgical treatment while the patient is in a stable clinical state rather than an emergency situation. DA is treated exactly the same whether it is associated with small intestinal atresia (SIA) or without. Duodenoduodenostomy remains the preferred management option for the treatment of duodenal atresia as well as Ladd's procedure for midgut malrotation. Our patient had been treated surgically with the aforementioned procedures. The prognosis for isolated situs inversus with an uneventful prenatal and postnatal course is excellent. However, when it is associated with other diseases, the prognosis can be impacted by that condition. Survival rates and long-term prognosis for DA are very good, too, at approximately 90% [18].

## Conclusions

The majority of the cases of situs inversus totalis are asymptomatic and are diagnosed incidentally during laparotomy or autopsy. When situs inversus totalis (SIT) is associated with DA, it presents early in the neonatal period. This association is extremely rare with a limited number of reported cases. Plain baby gram showing a reversed double bubble sign and reversed heart shadow is suggestive of DA with situs inversus. The diagnosis can be confirmed with a contrast meal and follow-through series. Screening of other possible associated anomalies is essential. Similar principles should be applied to surgical treatment as they are to classical cases and the mirror anatomy must be considered when performing the surgery. It is important for clinicians to have a high index of suspicion for malrotation with midgut volvulus or intestinal atresia in neonates of situs inversus presenting with bilious vomiting. Whether there are other anomalies or postoperative complications will determine the prognosis. The key to a favorable outcome is an early diagnosis and prompt referral. Considering the high significance of recognizing a rare condition, this case is noteworthy in our opinion.
